# The firstly visited department affects the acceptance of CPAP in patients with obstructive sleep apnea: a cohort study

**DOI:** 10.1186/s40463-023-00676-z

**Published:** 2023-10-28

**Authors:** Chung-Sheng Wu, David Hsin-Kuang Chen, Yuan-Chun Ko, Chyi-Huey Bai, Po-Yueh Chen, Wen-Te Liu, Yi-Chih Lin

**Affiliations:** 1 Department of Primary Care Medicine, Shuang-Ho Hospital, Taipei Medical University, New Taipei City, Taiwan; 2 Department of Medical Education, Shuang-Ho Hospital, Taipei Medical University, New Taipei City, Taiwan; 3 Department of Primary Care Medicine, Wan-Fang Hospital, Taipei Medical University, Taipei City, Taiwan; 4 School of Public Health, College of Public Health, Taipei Medical University, Taipei, Taiwan; 5 Department of Public Health, School of Medicine, College of Medicine, Taipei Medical University, Taipei, Taiwan; 6 Nutrition Research Center, Taipei Medical University Hospital, Taipei, Taiwan; 7 Department of Otolaryngology, School of Medicine, College of Medicine, Taipei Medical University, Taipei, Taiwan; 8 Department of Otolaryngology, Wan-Fang Hospital, Taipei Medical University, Taipei, Taiwan; 9 Sleep Center, Shuang-Ho Hospital, Taipei Medical University, New Taipei City, Taiwan; 10 Division of Pulmonary Medicine, Department of Internal Medicine, Shuang-Ho Hospital, Taipei Medical University, New Taipei City, Taiwan; 11 Department of Otolaryngology, Shuang-Ho Hospital, Taipei Medical University, No. 291, Zhongzheng Rd., Zhonghe District, New Taipei City, 23561 Taiwan

**Keywords:** Obstructive sleep apnea, Continuous positive airway pressure, Acceptance, Shared decision making

## Abstract

**Background:**

Continuous positive airway pressure (CPAP) therapy is the first-line treatment for obstructive sleep apnea (OSA). However, the low acceptance rate of CPAP remains a challenging clinical issue. This study aimed to determine the factors that influence the acceptance rate of CPAP.

**Methods:**

This retrospective cohort study was conducted at the sleep center of Shuang-Ho Hospital. Initially, 1186 OSA patients who received CPAP therapy between December 2013 and December 2017 were selected, and finally, 1016 patients were analyzed. All patients with OSA received CPAP therapy for at least 1 week, and their acceptance to treatment was subsequently recorded. Outcome measures included patients’ demographic and clinical characteristics (sex, age, BMI, comorbidities, history of smoking, and the medical specialist who prescribed CPAP treatment), polysomnography (PSG) results, and OSA surgical records.

**Results:**

Patients with a lower CPAP acceptance rate were referred from otolaryngologists (acceptance rate of otolaryngology vs. others: 49.6% vs. 56.6%, *p* = .015), in addition to having a lower apnea–hypopnea index (AHI) (acceptance vs. non-acceptance: 55.83 vs. 40.79, *p* = .003), rapid eye movement AHI (REM-AHI) (acceptance vs. non-acceptance: 51.21 vs. 44.92, *p* = .014), and arousal index (acceptance vs. non-acceptance: 36.80 vs. 28.75, *p* = .011). The multiple logistic regression model showed that patients referred from otolaryngology had a lower CPAP acceptance rate (odds ratio 0.707, *p* = .0216) even after adjusting for age, sex, BMI, AHI, REM-AHI, arousal index, comorbidities, and smoking status.

**Conclusions:**

Before their initial consultation, patients may already have their preferred treatment of choice, which is strongly linked to the type of medical specialists they visit, and consequently, affects their rate of acceptance to CPAP therapy. Therefore, physicians should provide personalized care to patients by exploring and abiding by their preferred treatment choices.

**Supplementary Information:**

The online version contains supplementary material available at 10.1186/s40463-023-00676-z.

## Background

Obstructive sleep apnea (OSA) is a chronic condition caused by the episodic collapse and obstruction of the upper airway during sleep. The prevalence of OSA syndrome ranges from 3 to 7% among adult men and 2–5% among adult women in the general population [1]. Among OSA patients in the United States, 17% of men and 9% of women are 50‒70 years of age [2]. OSA patients have a higher risk of daytime sleepiness; cardiovascular, neurovascular, and metabolic disturbances; motor vehicle accidents; and decreased psychomotor speed [3–8]; however, many of them remain undiagnosed [9].

Several treatment options are available, including lifestyle changes [10, 11], oral appliance [12], pharmacological treatment [13], surgical treatment [14, 15], and continuous positive airway pressure (CPAP) therapy. CPAP therapy can prevent upper airway collapse and is the first-line treatment for OSA owing to its immediate effects and relatively lower complication rates [16–18]. CPAP reduces daytime sleepiness [19–22], has beneficial effects in cardiovascular and metabolic diseases [23–28], and improves health-related quality of life, mood, and neurocognitive function, especially in subgroups with higher treatment adherence [19, 29].

The efficacy of CPAP therapy is correlated with patients’ adherence [30, 31]. Nevertheless, low acceptance and adherence to CPAP therapy remain challenging issues in clinical practice. According to Yang et al. and Simon-Tuval et al., the acceptance rate of CPAP is approximately 40% [32, 33]. CPAP adherence is often defined as using the therapy for an average of 4 h per night for at least 70% of the nights monitored [34]. Studies have shown that 46–83% of patients are non-adherent to treatment [35, 36]. Further, a systematic review of 82 studies found that CPAP non-adherence affects at least one-third of treated patients and showed that CPAP adherence did not significantly improve in the past 20 years despite efforts in behavioral intervention and patient coaching [37].

Many studies have focused on CPAP adherence and its correlated factors, and have shown inconsistent results with factors including age, sex, race, severity of OSA, severity of symptoms, smoking status, and socioeconomic status [38–46]. However, only a few studies have mentioned factors related to CPAP acceptance [32, 33, 47], and the specialties of doctors who prescribe CPAP treatment are rarely discussed. Achieving shared decision-making depends on building good relationships in clinical encounters, so it is important for physicians to understand patients and share information. Therefore, we aimed to investigate and analyze the factors that cause a lower acceptance rate of CPAP in patients with OSA.

## Methods

### Data collection and sample

This retrospective cohort study initially included 1186 OSA patients treated with CPAP at least 1 week at Shuang-Ho Hospital (New Taipei City, Taiwan) between December 2013 and December 2017. Patients with symptoms of OSA underwent polysomnography (PSG) at Shuang-Ho Hospital, and those diagnosed with OSA were referred to the hospital’s sleep center for CPAP treatment. Apart from otolaryngology, other outpatient departments, including pulmonology, cardiology, neurology, and psychiatry, also referred patients with OSA to the sleep center, and each patient received CPAP treatment for at least 1 week. In addition, patients were instructed on how to use the CPAP device correctly and followed up continuously for at least 3 months via telephone or mobile communication software by the sleep case manager of the sleep center to ensure adherence to the treatment regimen was achieved.

Eligibility criteria included patients aged 20 years or older, a record of CPAP use, and a diagnosis of OSA, which is defined as having an apnea–hypopnea index (AHI) of at least 5, based on the PSG records. Patients were excluded from the study if they were younger than 20 years of age, had an AHI of less than 5, had missing data on PSG records, or were referred from departments with fewer than 10 patient referrals to the sleep center.

The study was conducted in accordance with the guidelines of the Declaration of Helsinki and approved by the Institutional Review Board of Taipei Medical University (IRB Number: N202201092, 2022/4/14 of approval).

### Assessment of baseline demographic and clinical characteristics

Patients’ medical history and clinical characteristics were comprehensively reviewed through their admission, outpatient, and surgical records, including their demographic characteristics (age, sex, and BMI), specialties of their referral doctors, smoking status, and presence of baseline comorbidities, including hypertension (HTN), diabetes mellitus (DM), cardiovascular disease (CVD), chronic kidney disease (CKD), hyperlipidemia (HLD), and chronic obstructive pulmonary disease (COPD). Cardiovascular disease was defined as a coronary artery disease or cerebrovascular accident. For patients who did not receive CPAP therapy, we tracked whether they had undergone OSA-related surgeries. OSA-related surgery was defined as uvulopalatopharyngoplasty (UPPP), uvulopalatal flap surgery (UPF), laser-assisted uvulopalatoplasty (LAUP), pharyngoplasty, tonsillectomy, adenoidectomy, septoplasty, submucosal resection of the nose, and submucosal turbinectomy.

### Assessment of polysomnography results

PSG was conducted by certified respiratory therapists with at least 1 year of clinical experience. Standard monitoring devices included electroencephalography (EEG), electrooculography (EOG), electrocardiography (ECG), and electromyography (EMG) placed on the chin and bilateral anterior tibialis; position sensors, snore sensors, pulse oximetry, and thermistors to sense nasal and oral airflow; and plethysmography to sense thoracoabdominal movements.

PSG reports included the Epworth Sleepiness Scale (ESS) score, Pittsburgh Sleep Quality Index (PSQI), apnea–hypopnea index (AHI), rapid eye movement AHI (REM-AHI), non-rapid eye movement apnea–hypopnea index (NREM-AHI), sleep efficiency, mean oxygen saturation (mean SAT), mean heart rate (mean HR), arousal index, periodic limb movement index (PLMI), and pressure settings of the CPAP machine (90% pressure).

AHI was calculated as the number of apnea and hypopnea events per hour during sleep. REM-AHI was defined as the AHI during the rapid eye movement period. Sleep efficiency was defined as the ratio of the total sleep time to the total recording time. The 90% pressure was adjusted to eliminate 95% of apneas, hypopneas, and snoring to achieve an oxygen saturation of > 90% and AHI < 5 events/h in patients during REM sleep in a supine position.

### Acceptance and non-acceptance

All enrolled patients commenced CPAP therapy for at least 1 week as a test trial. Patients who purchased the CPAP machine were defined as the acceptance group, and those who did not were placed in the non-acceptance group. For those who accepted CPAP, we further documented the date of purchase and for those who did not accept, we recorded the date they returned the CPAP device.

### Statistical analyses

Continuous data were presented as mean ± standard deviation (SD) and tested by independent *t* test. Categorical data were presented as N (%) and tested by Chi-square test or Fisher's exact test. We further performed multivariate logistic regression to evaluate associations between factors chosen by this study and CPAP acceptance. Odds ratio (OR) and 95% confidence intervals (CI) were also calculated. Three models were established. Model 1 was adjusted by age, gender, and BMI. Model 2 was adjusted by covariates in Model 1, plus AHI, REM-AHI, and Arousal Index. Model 3 was adjusted by covariates in Model 2, plus HTN, DM, CVD, HLD, CKD, COPD, and smoking status. *P* value lower than 0.05 was regarded as statistically significant. Data were analyzed using SAS version 9.4 software (SAS Inc., Cary, NC, USA).

## Results

### Characteristics of the participants

**Fig. 1:**
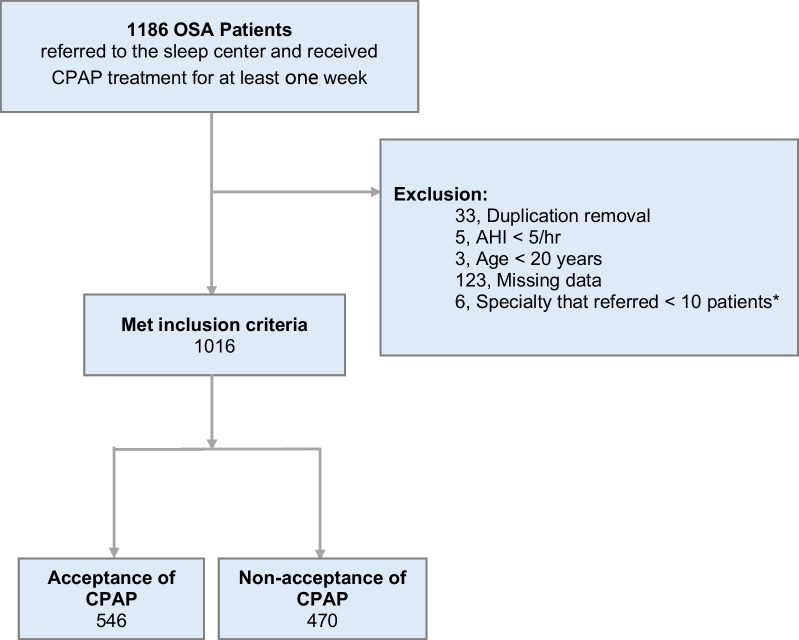
Flow diagram of included patients. *OSA* obstructive sleep apnea, *CPAP* continuous positive airway pressure, *AHI* apnea–hypopnea index. *Specialty referring to < 10 patients: 2 from nephrology, 4 from physical medicine and rehabilitation

A total of 1016 patients consisting of 825 men (81.2%) and 191 women (18.8%), mean age of 50.7 years, were enrolled for analysis (Fig. 1). A total of 546 (53.7%) patients accepted CPAP, and the baseline demographic and clinical characteristics of the acceptance group versus the non-acceptance group are shown in Table 1. We found that patients in the non-acceptance group were older (51.62 vs. 49.84, *p* = 0.031) and had lower body mass index (BMI) (28.54 vs. 29.33, *p* = 0.011). However, there were no significant differences in the baseline comorbidities between the two groups. Notably, the acceptance rate of CPAP therapy in patients referred by otolaryngologists was 49.6%, which was the lowest among all medical specialties who referred patients for CPAP treatment.

**Table 1:** Baseline demographic and clinical characteristics of the study participants

Characteristics	Acceptance N = 546	Non-acceptance N = 470	Univariate *p* value	Multivariate *p* value
Age, mean. (SD)	49.84 (12.9)	51.62 (13.2)	.031*	.143
Gender (male), %	83.3%	78.7%	.061	.114
BMI, mean. (SD)	29.33 (4.8)	28.54 (5.1)	.011*	.110
Smoking, %	31.9%	30.2%	.570	.814
Acceptance of specialties				
Surgeon, ENT, no. (%)	206 (49.6%)	209 (50.4%)	.029*	.015*
Non-surgeon, no. (%)	340 (56.6%)	261 (43.4%)		
PULMO no. (%)	209 (55.0%)	171 (45.0%)		
NEURO, no. (%)	76 (58.5%)	54 (41.5%)		
CV, no. (%)	41 (59.4%)	28 (40.6%)		
PSY, no. (%)	14 (63.6%)	8 (36.4%)		
Comorbidities				
HTN, %	45.6%	44.9%	.820	.756
DM, %	17.4%	16.8%	.803	.619
CVD, %	22.0%	20.9%	.663	.597
HLD, %	30.4%	28.1%	.418	.576
CKD, %	2.0%	3.6%	.120	.115
COPD, %	6.0%	7.0%	.529	.659

Univariate *p* value: *p* value without any variable adjustment. Multivariate *p* value: *p* value with adjustment of the others variables listed in the table

*SD* standard deviation, *BMI* body mass index, *no* number, *ENT* otolaryngology, *PULMO* pulmonology, *NEURO* neurology, *CV* cardiology, *PSY* psychiatry, *HTN* hypertension, *DM* diabetes mellitus, *CVD* cardiovascular disease, *HLD* hypertension, *CKD* chronic kidney disease, *COPD* chronic obstructive pulmonary disease

The overnight PSG results of the acceptance and non-acceptance groups are shown in Table 2. Multivariable analysis revealed that the non-acceptance group had a lower AHI (40.79 vs. 55.83, *p* = 0.003), REM-AHI (44.92 vs. 51.21, *p* = 0.014), and arousal index (28.75 vs. 36.80, *p* = 0.011).

**Table 2:** Baseline polysomnography results of the study participants

Polysomnography, mean. (SD)	Acceptance N = 546	Non-acceptance N = 470	Univariate *p* value	Multivariate *p* value
PSQ	9.47 (3.8)	9.30 (3.8)	.615	.264
ESS	11.26 (5.5)	10.64 (5.3)	.208	.507
AHI	55.83 (24.5)	40.79 (23.5)	< .001	.003*
REM-AHI	51.21 (25.3)	44.92 (25.6)	< .001	.014*
NREM-AHI	53.86 (27.3)	38.76 (24.8)	< .001	.781
90% PRESSURE	10.87 (2.6)	10.02 (2.4)	< .001	.201
MEAN SAT	94.06 (3.0)	94.95 (2.5)	< .001	.808
MEAN HR	70.22 (9.9)	68.63 (9.3)	.009	.842
AROUSAL INDEX	36.80 (22.6)	28.75 (19.2)	< .001	*.011
SLEEP EFFICIENCY	75.56 (17.1)	75.39 (16.8)	.878	.384
PLM	2.69 (9.0)	3.15 (10.7)	.461	.575

Univariate *p* value: *p* value without any variable adjustment. Multivariate *p* value: *p* value with adjustment of the others variables listed in the table

*SD* standard deviation, *PSQ* Pittsburgh Sleep Quality Index, *ESS* Epworth sleepiness scale, *AHI* apnea–hypopnea index, *REM-AHI* apnea–hypopnea index during rapid eye movement sleep, *NREM-AHI* apnea–hypopnea index during non-rapid eye movement sleep, *90% PRESSURE* average pressure measured during 90% of sleep, *MEAN SAT* mean blood oxygen saturation, *MEAN HR* mean heart rate, *PLM* periodic limb movement

Based on our results, the acceptance rate of CPAP therapy seemed to strongly correlate with the medical specialty that referred the patients for treatment. Hence, we performed a logistic model analysis to calculate the OR of otolaryngology patients who accepted CPAP (Table 3). In Model one, we adjusted the variables for baseline characteristics (sex, age, BMI). Model two was adjusted based on model one with the addition of PSG-related variables that significantly correlated with CPAP acceptance (AHI, REM-AHI, and arousal index). Model three was adjusted according to model two plus smoking status and presence of comorbidities (HTN, DM, CVD, HLD, CKD, and COPD). The OR was 0.707 (*p* = 0.0216, CI 0.526–0.950), which indicated that the odds of otolaryngology patients accepting CPAP treatment were significantly lower than those of non-otolaryngology patients, even after adjusting for all the factors above.

**Table 3:** Logistic model analysis to calculate the odds ratio of otolaryngology patients accepting CPAP

Variable	Odds ratio	*p* value	95% CI
Model 1	0.715	.0128	0.550‒0.931
Model 2	0.721	.0249	0.542‒0.960
Model 3	0.707	.0216	0.526‒0.950

Model 1 was adjusted for age, gender, BMI

Model 2 was adjusted for Model 1, AHI, REM-AHI and Arousal Index

Model 3 was adjusted for Model 2, HTN, DM, CVD, HLD, CKD, COPD and smoking

*CI* confidence interval

### Analysis of otolaryngology versus other departments

Since patients referred from otolaryngology had a lower CPAP acceptance rate, we aimed to further analyze the differences between these patients and those referred from other medical specialties. The baseline demographic and clinical characteristics are shown in Table 4. We found that patients from the otolaryngology department were younger (46.97 vs. 53.22, *p* < 0.001), had lower BMI (28.49 vs. 29.29, *p* = 0.012), were more likely to be male (84.6% vs. 78.9%, *p* = 0.022), and had fewer smokers (24.8% vs. 35.4%, *p* < 0.001). Compared with other medical specialties, patients referred from otolaryngology also had significantly less comorbidities such as hypertension (30.4 vs. 55.6%, *p* < 0.001), diabetes mellitus (12.3% vs. 20.5%, *p* < 0.001), cardiovascular diseases (11.8% vs. 28.1%, *p* < 0.001), hyperlipidemia (21.9% vs. 34.4%, *p* < 0.001), and chronic obstructive pulmonary disease (1.2% vs. 10.1%, *p* < 0.001).

**Table 4:** Demographic and clinical characteristics of patients from otolaryngology versus other specialties

Characteristics	Otolaryngology N = 415	Others N = 601	Univariate *p* value
Age, mean (SD)	46.97 (11.8)	53.22 (13.3)	< .001
Gender (male), %	84.6%	78.9%	.022
BMI, mean (SD)	28.49 (5.0)	29.29 (4.9)	.012
Smoking, %	24.8%	35.4%	< .001
*Comorbidities*			
HTN, %	30.4%	55.6%	< .001
DM, %	12.3%	20.5%	< .001
CVD, %	11.8%	28.1%	< .001
HLD, %	21.9%	34.4%	< .001
CKD, %	1.9%	3.3%	.180
COPD, %	1.2%	10.1%	< .001

*SD* standard deviation, *BMI* body mass index, *HTN* hypertension, *DM* diabetes mellitus, *CVD* cardiovascular disease, *HLD* hyperlipidemia, *CKD* chronic kidney disease, *COPD* chronic obstructive pulmonary disease

The overnight PSG results of patients referred from otolaryngology versus patients referred from other specialties are shown in Table 5. We observed that patients referred from otolaryngology exhibited no significant difference in OSA severity compared to patients referred by other specialties, including variables that determine CPAP acceptance, such as AHI (*p* = 0.299), REM-AHI (*p* = 0.855), and arousal index (*p* = 0.080).

**Table 5:** Polysomnography results of patients from otolaryngology versus other specialties

Polysomnography, mean (SD)	Otolaryngology N = 415	Others N = 601	Univariate *p* value
PSQ	9.14 (3.6)	9.60 (3.9)	.194
ESS	10.76 (5.3)	11.13 (5.5)	.456
AHI	47.89 (26.1)	49.56 (24.5)	.299
REM-AHI	48.11 (25.7)	48.41 (25.6)	.855
NREM-AHI	47.36 (27.7)	46.54 (26.8)	.634
90% PRESSURE	10.37 (2.5)	10.55 (2.5)	.258
MEAN SAT	94.69 (2.8)	94.32 (2.8)	.039
MEAN HR	69.22 (9.6)	69.67 (9.7)	.469
AROUSAL INDEX	34.52 (22.8)	32.07 (20.5)	.080
SLEEP EFFICIENCY	77.08 (16.2)	74.38 (17.4)	.013
PLM	2.32 (8.7)	3.31 (10.5)	.102

*SD* standard deviation, *PSQ* Pittsburgh Sleep Quality Index, *ESS* Epworth Sleepiness Scale, *AHI* apnea–hypopnea index, *REM-AHI* apnea–hypopnea index during rapid eye movement sleep, *NREM-AHI* apnea–hypopnea index during non-rapid eye movement sleep, *90% PRESSURE* average pressure measured during 90% of sleep, *MEAN SAT* mean blood oxygen saturation, *MEAN HR* mean heart rate, *PLM* periodic limb movement

From the analysis of the population accepting CPAP, it was found that patients referred by the otolaryngology department had a higher proportion of seeking surgical intervention compared to other departments. This difference is statistically significant, with percentages of 19.2% versus 7.5%, respectively (*p* < 0.001).

**Fig. 2:**
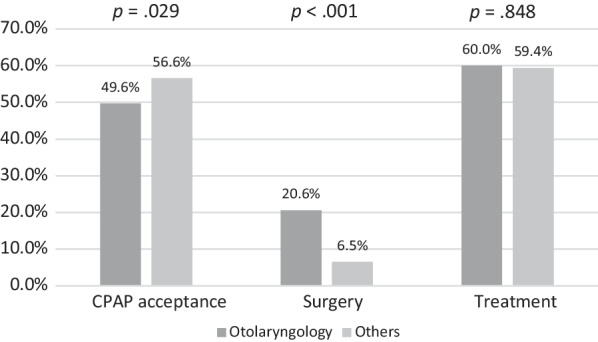
Ratios of accepting CPAP, receiving surgery, and acquiring treatment in otolaryngology patients versus other specialties. *CPAP* continuous positive airway pressure

We further analyzed whether patients underwent OSA surgery after refusing CPAP therapy and discovered that a higher ratio of patients from otolaryngology underwent OSA surgery (20.6% vs. 6.5%, *p* < 0.001, Fig. 2). Subsequently, we combined the data of patients who underwent CPAP or OSA surgery as receiving treatment for OSA and found no significant difference between the two groups (Fig. 2).

According to the baseline demographic characteristics and PSG results of the patients who underwent OSA surgery, there was no significant difference between the groups (Table 6).

**Table 6:** Demographic characteristics and polysomnography results of patients receiving surgery after refusing CPAP therapy

	Otolaryngology N = 43	Others N = 17	Univariate *p* value
Characteristics			
Age, mean (SD)	46.86 (11.08)	50.76 (12.58)	.241
Gender (male), %	81.4%	70.6%	.570
BMI, mean (SD)	28.32 (5.54)	28.70 (5.82)	.813
Smoking, %	27.9%	47.1%	.156
HTN, %	41.9%	47.1%	.714
DM, %	18.6%	5.9%	.400
CVD, %	14.0%	23.5%	.608
HLD, %	20.9%	41.2%	.203
CKD, %	2.3%	0.0%	.628
COPD, %	0.0%	0.0%	nil
Polysomnography, mean (SD)			
PSQ	9.71 (3.94)	8.86 (2.34)	.593
ESS	10.50 (5.75)	10.43 (5.26)	.977
AHI	46.19 (25.19)	39.06 (21.28)	.308
REM-AHI	54.50 (29.46)	44.61 (27.81)	.277
NREM-AHI	44.99 (27.08)	35.33 (23.88)	.204
90% PRESSURE	10.12 (2.63)	10.06 (2.28)	.929
MEAN SAT	94.92 (1.82)	94.75 (2.60)	.776
MEAN HR	71.26 (10.37)	70.91 (9.62)	.906
AROUSAL INDEX	34.24 (22.54)	24.61 (14.82)	.110
SLEEP EFFICIENCY	74.79 (16.51)	71.12 (21.97)	.484
PLM	3.83 (17.00)	4.94 (14.27)	.814

*SD* standard deviation, *BMI* body mass index, *HTN* hypertension, *DM* diabetes mellitus, *CVD* cardiovascular disease, *HLD* hyperlipidemia, *CKD* chronic kidney disease, *COPD* chronic obstructive pulmonary disease, *PSQ* Pittsburgh Sleep Quality Index, *ESS* Epworth Sleepiness Scale, *AHI* apnea–hypopnea index, *REM-AHI* apnea–hypopnea index during rapid eye movement sleep, *NREM-AHI* apnea–hypopnea index during non-rapid eye movement sleep, *90% PRESSURE* average pressure measured during 90% of the sleep, *MEAN SAT* mean blood oxygen saturation, *MEAN HR* mean heart rate, *PLM* periodic limb movement

## Discussion

The present study showed that OSA patients with lower CPAP acceptance tend to be referred to the sleep center by otolaryngologists and had milder OSA severity (specifically lower AHI, REM-AHI, and arousal index), lower BMI, and older age. After excluding patients who initially visited the otolaryngology department, we found that patients from other specialties exhibited similar trends to the original data (Additional file 1: Table S1 and Table S2).

A total of 53.7% (546/1016) of patients accepted CPAP in the present study, which was substantially higher compared to the 40.1% in Simon et al. (n = 162, mean age 54.9 y/o) and 39.7% in Yang et al. (n = 315, mean age 56.7 y/o). This may be attributed to the younger age (50.7 y/o) of our cohort. Furthermore, instructions and assistance were provided to patients by the case manager of the sleep center via a mobile communication software on the correct use of the device in order to maximize their acceptance of CPAP treatment.

Previous studies have consistently demonstrated that greater OSA severity is associated with higher CPAP adherence [33, 38, 39, 42–44, 46, 48]. However, demographic characteristics, including sex, age, BMI, comorbidities, and smoking, showed inconsistent results [45]. In addition, we found that BMI and age significantly influenced acceptance of CPAP. Several studies have shown that higher ESS scores and more severe OSA symptoms are associated with higher CPAP adherence [33, 39–41, 44, 46]. Our results displayed similar trends but without statistical significance. Moreover, it is noteworthy that comorbidities and PLMI, which have not been discussed in previous studies, did not appear to be significant predictors of acceptance of CPAP.

We further compared the demographic and clinical characteristics of patients referred by otolaryngologists with those of patients referred from other specialties. Patients referred from otolaryngology had a lower BMI, fewer comorbidities, fewer smokers, and more young men; however, the PSG results were similar between the two groups (Tables 4, 5). According to previous studies, OSA severity is highly correlated with CPAP acceptance [33, 38, 39, 42–44, 46, 48]. Nevertheless, our study showed that otolaryngology patients had a lower acceptance of CPAP but demonstrated the same OSA severity as patients from other specialties (Table 5).

Similar to our results, Salas et al. showed that patients referred from otolaryngology had fewer comorbidities, lower BMI, and younger age, except for having the same OSA severity as other specialties [49]. Furthermore, after adjusting for multiple variables that may affect the CPAP acceptance rate, such as age, BMI, AHI, REM-AHI, and arousal index, the OR of CPAP acceptance in patients from otolaryngology was still significantly lower than that in patients from other departments (OR = 0.707, *p* = 0.0216, Table 3).

Three possible reasons may explain the disparity in patients’ choice of medical specialties for the treatment of OSA: the intention for surgery, surgical risks involved, and knowledge about the treatment.

First, our study showed that patients from otolaryngology who did not accept CPAP showed higher incidences of undergoing surgery afterwards than non-otolaryngology OSA patients (20.6% vs. 6.5%, *p* < 0.001, Fig. 2). Furthermore, we analyzed the ratio of OSA-related treatments, including CPAP or OSA surgery, and found no significant difference between patients from otolaryngology and those from other departments (*p* = 0.848, Fig. 2). This may indicate that both groups shared a similar intent for treatment, and a substantial portion of otolaryngology patients who refused CPAP treatment may have undergone surgery. In addition, we analyzed the AHI of the patients who underwent surgery and found no significant difference between the groups (*p* = 0.308, Table 6). This demonstrates that the two groups had similar indications for OSA surgery. In summary, patients’ intention to undergo surgery may contribute to their preference for otolaryngology clinics when seeking treatment for OSA. Second, our results were consistent with those of Salas et al., which showed that patients from otolaryngology had a lower risk of general anesthesia and surgical treatment [49]. Hence, we infer that patients’ surgical risks may affect their preference for specialties when they seek treatment. Third, previous studies showed that patients’ knowledge and beliefs about the disease affected their adherence to treatment [36, 50, 51]. Gulati et al. [52] pointed out that patients receiving surgery for OSA, compared to those who did not, had a higher ratio of independently doing research for OSA treatment prior to their consultation with the surgeon. This would explain why patients’ knowledge about the variety of treatment options plays a crucial role in their adherence and decision-making, which may also determine their choice of medical specialties.

Patients who visit different departments may have different treatment preferences for OSA. According to the principle of shared decision making [53, 54], increasing patient participation in discussions on treatment options may provide a better therapeutic effect.

Our study has several advantages. First, to the best of our knowledge, this is the first study to investigate the rate of acceptance of CPAP in patients referred by different specialties. Second, our sample size was relatively large (n = 1016) compared to previous studies. Third, we selected a more stringent definition of CPAP acceptance in our study, where “Acceptance” was defined as patients who purchased CPAP machines after their test trial, which is also the same definition adopted by Yang and Simon-Tuval [32, 33]. Compared to Rauscher et al., which only had a 3-day CPAP test trial [47], our definition of CPAP acceptance may better reflect the patients’ actual intent.

This study has several limitations. First, this was a retrospective cohort study; therefore, potential participants were excluded because of missing data, and the baseline data of the otolaryngology versus non-otolaryngology groups may be different. Second, few patients were referred from certain medical specialties. For example, patients from the Department of Psychiatry had a higher acceptance of CPAP compared to other specialties; however, no statistical significance was noted. Lastly, some patients in the non-acceptance group may purchase CPAP via alternative routes; as a result, this may have caused a selection bias. In order to minimize this bias, the medical records of the non-acceptance group were carefully examined during their subsequent visits to the hospital to ensure that no CPAP treatment had been initiated. Moreover, patients from the non-acceptance group were constantly followed up by the case manager of the sleep center regarding their subsequent treatment for OSA, and whether they received treatment elsewhere or if they were using other respiratory devices.

## Conclusions

Shared decision making is crucial for achieving therapeutic success in clinical practice. In the sleep center, patients with OSA who initially visited otolaryngology clinics had a lower acceptance rate for CPAP. Therefore, besides adhering to clinical guidelines, physicians should provide individualized care through shared decision-making and identifying patients’ preferred treatment.

### Supplementary Information

**Additional file 1.** Baseline Data for Study Participants Referred by Non-Surgeons.

## Data Availability

The datasets analyzed in the current study are available from the corresponding author upon reasonable request.

## Abbreviations

AHI: Apnea–hypopnea index
BMI: Body mass index
CI: Confidence intervals
CKD: Chronic kidney disease
COPD: Chronic obstructive pulmonary disease
CPAP: Continuous positive airway pressure
CVD: Cardiovascular disease
DM: Diabetes mellitus
ECG: Electrocardiography
EEG: Electroencephalography
EMG: Electromyography
EOG: Electrooculography
ESS: Epworth sleepiness scale
HLD: Hyperlipidemia
HR: Heart rate
HTN: Hypertension
IRB: Institutional Review Board
LAUP: Laser-assisted uvulopalatoplasty
NREM-AHI: Non-rapid eye movement apnea–hypopnea index
OR: Odds ratio
OSA: Obstructive sleep apnea
PLMI: Periodic limb movement index
PSG: Polysomnography
PSQI: Pittsburgh Sleep Quality Index
REM-AHI: Rapid eye movement AHI
SAT: Saturation
SD: Standard deviation
UPF: Uvulopalatal flap surgery
UPPP: Uvulopalatopharyngoplasty
