# Supplementary material for: The firstly visited department affects the acceptance of CPAP in patients with obstructive sleep apnea: a cohort study
Source: J Otolaryngol Head Neck Surg. 2023 Oct 28;52:71. doi: 10.1186/s40463-023-00676-z (PMC10613393; doi:10.1186/s40463-023-00676-z)
Supplement: Supplementary file 1 — Additional file 1. Baseline Data for Study Participants Referred by Non-Surgeons. [file 40463_2023_676_MOESM1_ESM.docx]

**Table S1. Baseline demographic and clinical characteristics of the study participants referred from non-surgeon**

|  | Acceptance | | Non-acceptance | |  |  |
| --- | --- | --- | --- | --- | --- | --- |
|  | Mean/% | SD | Mean | SD | Univariate P value | Multivariate P value |
| Gender | 80.59% | - | 76.63% | - | 0.239 | - |
| Age | 54.32 | 13.08 | 54.38 | 13.56 | 0.06 | 0.219 |
| BMI | 29.65 | 4.84 | 28.83 | 4.97 | 0.043 | 0.150 |
| HTN | 56.18% | - | 54.79% | - | 0.734 | - |
| DM | 20.88% | - | 19.92% | - | 0.773 | - |
| CVD | 28.82% | - | 27.20% | - | 0.661 | - |
| HLD | 35.00% | - | 33.72% | - | 0.743 | - |
| CKD | 2.65% | - | 4.21% | - | 0.288 | - |
| COPD | 8.82% | - | 11.88% | - | 0.219 | - |
| smoke | 34.71% | - | 36.40% | - | 0.667 | - |

1. SD, standard deviation; BMI, body mass index; HTN, hypertension; DM, diabetes mellitus; CVD, cardiovascular disease; HLD, hypertension; CKD, chronic kidney disease; COPD, chronic obstructive pulmonary disease.
2. Univariate p value: P value without any variable adjustment. Multivariate p value: P value with adjustment of the significant variables from univariate analysis listed in the table.

**Table S2. Baseline polysomnography results of the study participants referred from non-surgeon**

|  | Acceptance | | Non-acceptance | |  |  |
| --- | --- | --- | --- | --- | --- | --- |
|  | Mean | SD | Mean | SD | Univariate P value | Multivariate P value |
| PSQ | 9.61 | 3.89 | 9.58 | 4.02 | 0.961 | - |
| ESS | 11.51 | 5.65 | 10.65 | 5.25 | 0.212 | - |
| AHI | 56.41 | 23.81 | 40.63 | 22.51 | <0.001 | *0.001 |
| REMAHI | 51.63 | 25.49 | 44.21 | 25.15 | 0.001 | 0.333 |
| NREMAHI | 53.43 | 27.06 | 37.56 | 23.68 | <0.001 | 0.262 |
| pressure | 11.02 | 2.57 | 9.94 | 2.38 | <0.001 | 0.261 |
| MeanSAT | 93.90 | 3.04 | 94.86 | 2.47 | <0.001 | 0.574 |
| meanHR | 70.67 | 9.96 | 68.35 | 9.21 | 0.004 | 0.257 |
| ArousalIndex | 35.66 | 22.00 | 27.4 | 17.22 | <0.001 | 0.978 |
| Sleepeff | 75.08 | 17.14 | 73.47 | 17.74 | 0.262 | - |
| PLM | 3.11 | 9.36 | 3.57 | 11.89 | 0.604 | - |

1. SD, standard deviation; PSQ, Pittsburgh Sleep Quality Index; ESS, Epworth sleepiness scale; AHI, apnea-hypopnea index; REM-AHI, apnea-hypopnea index during rapid eye movement sleep; NREM-AHI, apnea-hypopnea index during non-rapid eye movement sleep; 90% PRESSURE, average pressure measured during 90% of sleep; MEAN SAT, mean blood oxygen saturation; MEAN HR, mean heart rate; PLM, periodic limb movement.
2. Univariate p value: P value without any variable adjustment. Multivariate p value: P value with adjustment of the significant variables from univariate analysis listed in the table.
